# Viable placental allograft as a biological dressing in the clinical management of full-thickness thermal occupational burns: Two case reports

**DOI:** 10.1097/MD.0000000000009045

**Published:** 2017-12-08

**Authors:** Eric L. Johnson, Elisabet K. Tassis, Georgina M. Michael, Susan G. Whittinghill

**Affiliations:** a Bozeman Deaconess Hospital, Wound and Hyperbaric Center, Bozeman, Montana; b Osiris Therapeutics, Inc., Columbia, Maryland.

**Keywords:** amniotic membrane, full-thickness wound, occupational injury, thermal burn, viable cryopreserved placental allograft

## Abstract

**Rationale:**

Occupational burn injuries can be detrimental and difficult to manage. The majority of complex cases are referred and managed at regional burn centers where access to specialized care is available. As an alternative to hospitalization with staged surgical procedures, placental products may be used for outpatient medical management of these common burn injuries, especially if access to a regional burn center is limited or restricted.

Fresh amnion has been a treatment of choice in burns for more than 100 years. As a biological covering with a broad scope of potential uses, human placental membranes represent a dressing that is particularly advantageous for burn therapy. Recent advances in tissue-preservation technology have allowed for the commercialization of placental amnion products.

**Patient concerns:**

To address several complications associated with burn injuries—contractures, scar formation, and pain—a viable cryopreserved placental membrane (vCPM) (Grafix—PRIME, Osiris Therapeutics, Inc., MD) retaining the anti-inflammatory, anti-fibrotic, and antimicrobial properties of fresh placental tissues was chosen for clinical use in the 2 cases reported, where both patients had restricted access to the regional burn center.

**Diagnoses:**

Two cases of work-related extremity burns presented to a local rural hospital for immediate post-injury assessment. The 1^st^ case was of a man who sustained a 55.4 cm^2^ full-thickness 3^rd^ degree thermal burn with exposed bone and tendon, to the left dorsal forefoot after having an industrial pressure washer caught on his work boot. The 2^nd^ case was of a female who sustained a 4.7 cm^2^ full-thickness 3^rd^ degree crush burn to the dorsum extensor surface of her dominant hand's index finger after applying 80-pounds per square inch of heated pressure from a hydraulic press.

**Interventions:**

Both burn patients elected to continue their care at the outpatient-based wound and hyperbaric center, receiving a combination of weekly ad libitum debridement, applications of vCPM, and occupational therapy.

**Outcomes:**

Both burns reached timely wound closure, and patients regained full range of motion of the affected limb, allowing for early return to work. The average number of allograft applications was 7.5, allowing both patients to return to work in an average of 63.5 days without adverse events or post-treatment complications.

**Lessons:**

The incorporation of this product in the treatment of these complex burns prevented amputation in one patient, and skin autografting and potential index finger contracture-formation in the second patient. The incorporation of vCPM in burn management may offer a new approach to outpatient burn management and may mitigate several of the complications seen post burn injury, leading to favorable patient outcomes.

## 1 Introduction

More than 40% of all medically treated burns are a result of occupationally based accidents.^[1]^ Work-related hazards give way to nonfatal combination injuries in which the affected limb may suffer both thermal- and crush- or high-pressure injection-related tissue damage.^[2]^ Partial and full-thickness burn injuries to the upper and lower extremities are the most common injuries and require early post-injury care to avoid functional impairments resulting from contracture or amputation.^[1,2]^ Individuals without geographic access to a regional burn center may rely on emergency department assessments with outpatient referral for definitive treatment and rehabilitation.^[3]^ In this setting, as an alternative to hospitalization with staged xenografting or dermal substitutes followed by autografting, initial surgical debridement alongside biological dressings such as human placental membranes (HPMs) may provide a viable option for outpatient medical management of these common burn injuries.

HPMs—mostly fresh human amnion—have been a treatment of choice in burn management for >100 years.^[4–7]^ Specifically, in the last 2 decades, the clinical benefits of amnion have been documented for burn management, infection control, pain reduction, and thermoregulation maintenance, and numerous preservation methods of amnion have been explored.^[7–10]^ Advances in tissue-preservation technology have led to the development of various point-of-care amniotic biological coverings that, when applied, serve as an adherent dressing that protects the wound while maintaining a moist environment, reduce pain, and support epithelialization.^[11]^ Amniotic membranes also provide antimicrobial activity that protects the area from secondary infection, a major contributor to complication, and poor burn-wound resolution.^[12,13]^

**Figure 1:**
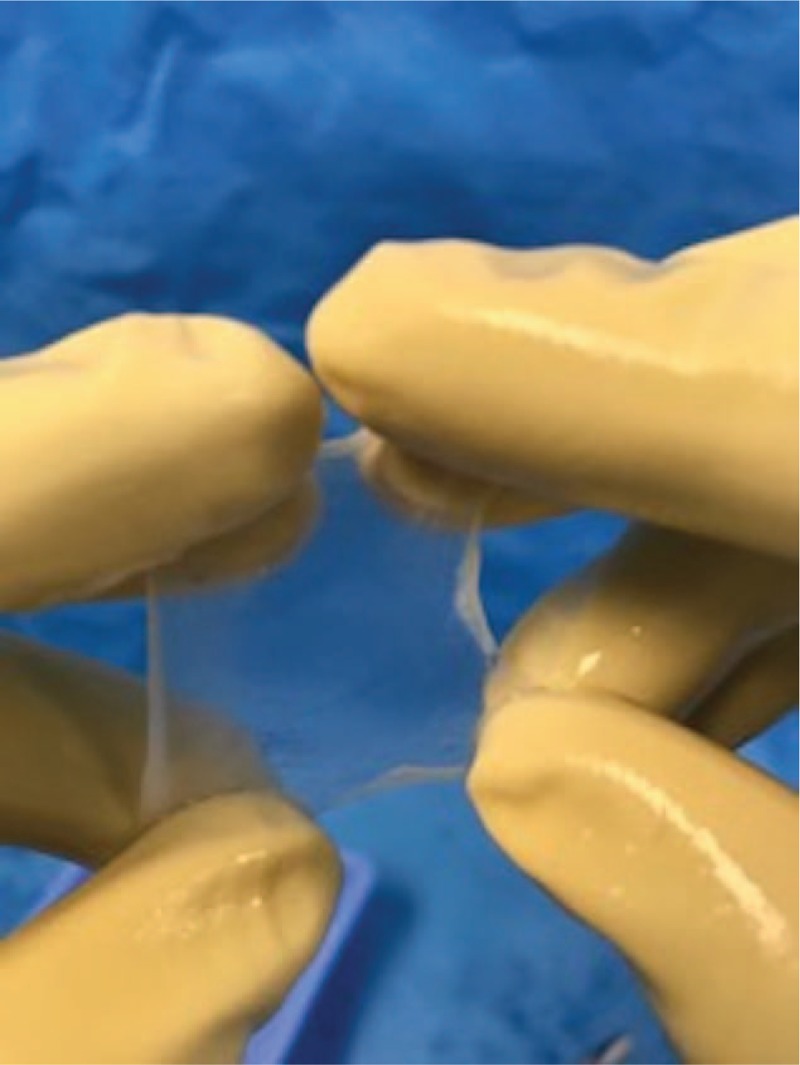
Thawed vCPM (3 × 4 cm) ready for point-of-care application to burn-wound bed. vCPM = viable cryopreserved placental membrane.

The antimicrobial, analgesic, proangiogenic, and anti-inflammatory properties of HPMs are mediated by components in the tissue that may be altered or destroyed during tissue processing and preservation.^[12]^ Viable cryopreserved placental membrane (vCPM) (Grafix—PRIME, Osiris Therapeutics, Inc., MD) is a commercially available allograft processed with an aseptic cryopreservation technology that retains the native HPM components: a structural 3-D ECM, resident growth factors and cytokines (PDGF, bFGF, EGF, KGF, VEGF, TGF-β3, EGF), and viable cells, including neonatal MSCs, epithelial cells, and fibroblasts. vCPM retains the anti-inflammatory, anti-fibrotic, chemoattractive, pro-angiogenic, and antimicrobial properties of fresh tissue that are known to be beneficial for deep thermal-burn treatment.^[14–16]^ This point-of-care allograft is stored frozen at –80 °C with 2-years shelf life.^[17]^ The membranes retain their structure after thawing and can be easily handled and applied (Fig. 1).

Here, we present 2 cases of work-related extremity burns presenting to a local rural hospital for immediate post-injury assessment. The nearest burn care facility exceeded 6 hours of travel time by car with a distance of 412 miles. Both patients elected to continue their care at the outpatient-based wound and hyperbaric center, receiving a combination of weekly ad libitum debridement, applications of vCPM, and occupational therapy (OT).

## 2 Case reports

### 2.1 Patient 1: Full-thickness 3^rd^ degree burn via high-pressure heated water injection to the foot

**Figure 2:**
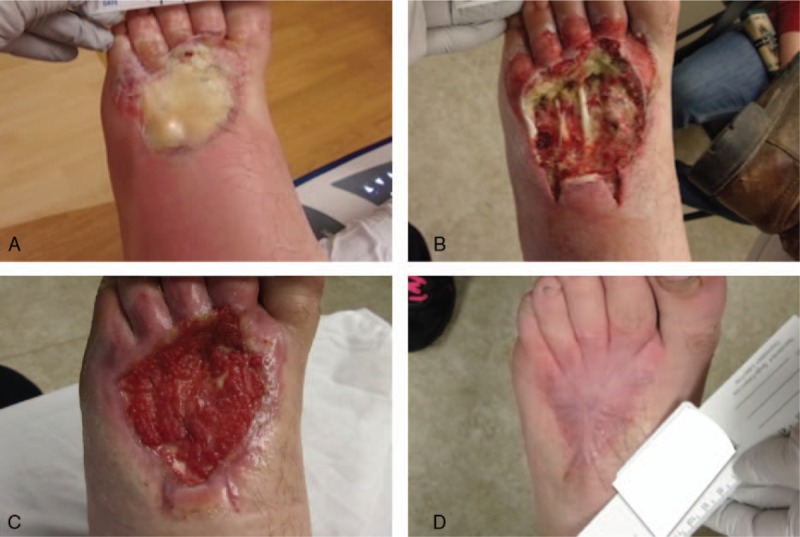
Full-thickness burn via high-pressure heated water injection to the left dorsal forefoot at (A) emergency room evaluation immediately following injury, (B) postsurgical debridement, (C) after demonstration of progressive granulation, and (D) full burn-wound closure.

A healthy 23-year-old man presented to the emergency department after an industrial pressure washer was caught on his work boot, resulting in a 1% total body-surface area high-pressure heated water injection injury to the lower extremity. Upon initial assessment, the dorsal aspect of the left forefoot was warm to the touch with evident cellulitis. The area of burned tissue was demarcated by white discoloration and blistering with clear drainage (Fig. 2A). Patient was negative for fever or chills and reported a pain score of 5/10 upon admission. After intravenous administration of cefazolin (2 g), the patient was transferred to the operating room for surgical irrigation and debridement.

In the operating room, copious pulse lavage was performed with 2 L of sterile saline containing 100,000 units of bacitracin to irrigate the wound. After careful excision of devitalized and burned tissues, wound-surface area was 55.4 cm^2^ with exposed bone and extensor tendons of the left dorsal forefoot **(**Fig. 2B). Initial wound dressings over exposed vital structures included bacitracin and a nonadherent layer (Adaptic, Acelity, TX) secured with a negative-pressure dressing and supportive elastic bandage wrap. Purulent discharge from the wound was cultured positive for bacillus at postoperative day 4. The patient was prescribed cephalexin (500 mg 4 times per day) for the infection and received surgeon referral to the hospital-based wound and hyperbaric center for continuation of care.

Beginning post burn-injury day 15, the patient began receiving weekly outpatient evaluation and care that included episodic debridement and compression and serial grafting with vCPM. When necessary, sharp excision of nonviable tissue was performed with tissue forceps and scissors after administration of 4% topical lidocaine. Placental grafts were covered with a nonadherent layer (Mepitel, Mölnlycke Health Care, Sweden) followed by a negative-pressure bolster. Negative-pressure dressings were discontinued at day 44 and transitioned to a 2-layer bandage compression system (Comperm, Hartmann USA, Inc., SC) with every-other-day secondary dressing changes using bacitracin ointment, collagen, and petroleum jelly gauze. Ongoing vCPM applications contributed to progressive granulation over exposed structures **(**Fig. 2C). There were no further episodes of secondary-burn infection. Complete burn-wound resolution resulted in lower limb salvage after a total of 10 vCPM applications **(**Fig. 2D). The patient returned to work at post-injury day 93 with a pain score of 0/10.

### 2.2 Patient 2: Full-thickness 3^rd^ degree crush burn to the finger via heated hydraulic press

**Figure 3:**
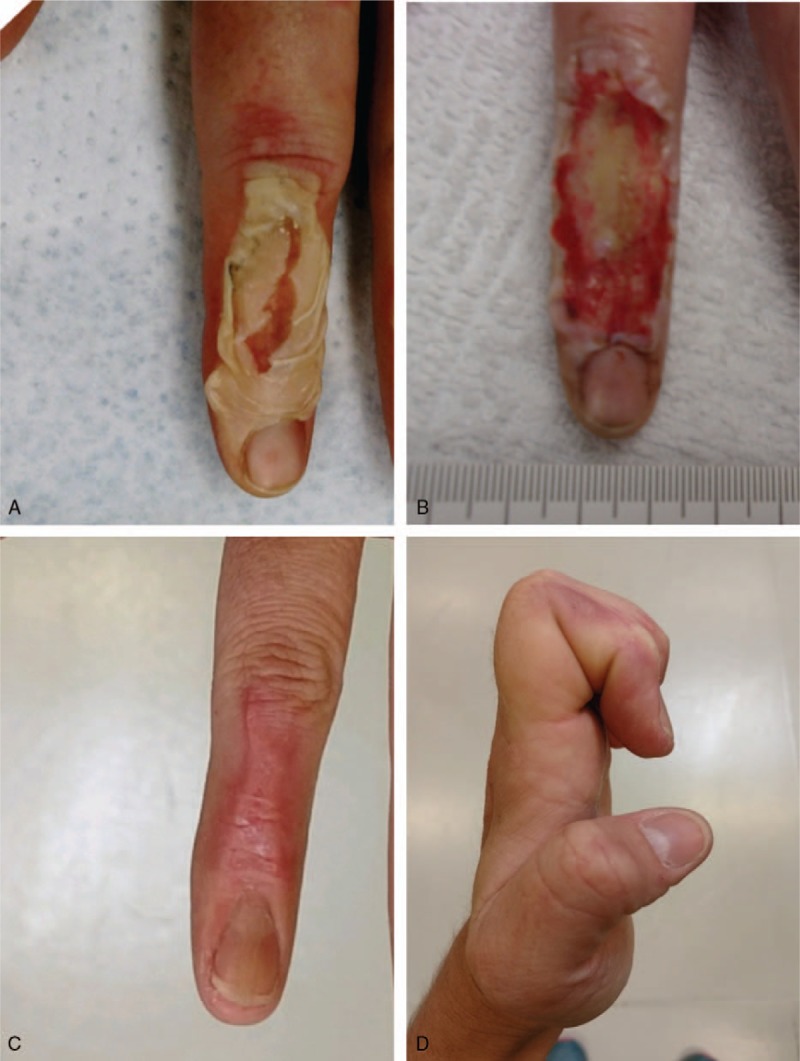
Full-thickness crush burn to the left index finger via heated hydraulic press following compression with 80 psi at (A) emergency room evaluation immediately following injury, (B) post bedside debridement, (C) complete reepithelialization, and (D) during follow-up demonstrating functional range of motion.

A healthy 30-year-old woman presented to the emergency department after a digit of her dominant hand was caught in hydraulic high-temperature machinery where 80-pounds per square inch (psi) of heated pressure were applied for nearly 10 seconds. The machinery caused a <1% total body surface area 4.7 cm^2^ full-thickness burn injury to the dorsum extensor surface of the left index finger with involvement of the middle interphalangeal joint and a portion of the proximal nailbed. Approximately, 50% of the region was blanched and surrounded by erythema without drainage **(**Fig. 3A). A consulting surgeon performed bedside-emergency sharp debridement of the nonviable burned tissue (Fig. 3B). Silver sulfadiazine (Silvadene, Pfizer, Inc., NY) was applied to the burn and a basic primary nonadherent bandage was applied.

At 2 days post injury, the patient received evaluation at the outpatient wound and hyperbaric center where a telemedicine consultation with the nearest regional burn facility was held. Burn surgeon recommendation consisted of staged surgical care with application of a dermal regeneration template followed by split thickness skin autografting. Alternatively, the patient chose to receive serial outpatient grafting with vCPM in addition to sessions of hyperbaric oxygen therapy at 2.4 atmospheres absolute (ATA). Grafts were covered with a nonadherent layer (Mepitel, Mölnlycke Health Care, Sweden) followed by a light compressive bandage. As the burn resolution progressed, OT was initiated where active range of motion (ROM) values were obtained and tracked, demonstrating progressive gains in functional mobility over time. The patient-reported level of pain with initiation of OT was 4/10, which then decreased to 0/10 prior to returning to work at day 34 post injury. A total of 5 vCPM applications contributed to burn-wound resolution without debilitating contracture of the digit (Fig. 3C). There were no incidents of burn infection reported. Upon follow-up, the patient expressed gratitude after regaining full ROM of her interphalangeal joint and was able to resume playing the guitar (Fig. 3D**)**.

## 3 Discussion

Burn injuries presenting to the emergency department can be managed at the local level, yet more extensive burn injuries that involve the hand, foot, surface over a joint, or affect >5% total body surface area are generally referred and managed at the nearest burn center with access to specialized care.^[18–20]^ In these 2 cases, despite referral to a burn unit, treatment was successfully conducted at the local wound clinic with the use of HPMs. Both patients attained burn-wound closure with an average 7.5 vCPM applications and ultimately returned to work in a mean time of 63.5 days.

Research indicates that, on average, only 66% of patients return to work following burn injury.^[21]^ Schneider et al^[22]^ reported that impaired mobility, defined as the presence of contractures or amputation, was the only statistically significant predictor of unemployment post burn injury at the 12-month mark. Initially, given the severity of the burn injury with exposed bone and tendon, amputation of the foot was considered for patient 1. However, the use of vCPM offered an alternative to amputation allowing the patient to regain lower limb function and normal ambulation. This is consistent with previously published data where similarly complex wounds were treated with vCPM and subsequently healed, avoiding the need for amputation.^[23,24]^ In the case of patient 2, the severity of the crush-burn injury placed the patient at high risk for long-term functional loss of the index finger due to contracture over the joint. A telemedicine consultation with a regional burn center included recommendations for staged autografting with a dermal regeneration template. Poor ROM was demonstrated during the initial assessment of all index finger joints. Following treatment with vCPM and OT, significantly improved active ROM values were obtained for patient 2. Final ROM measurements were comparable with the uninjured finger allowing full flexion of the joint (Fig. 3D).

Several studies have also indicated that a patient's return to work time is prolonged in the presence of ongoing pain.^[22,25,26]^ Following treatment with vCPM, both patients in this report demonstrated a measureable reduction in pain scores from the time of admittance to the time of returning to work. This observation is consistent with other published data indicating pain reduction after treatment with amnion.^[27–29]^ Both patients were prescribed narcotics, a practice often seen in burn-pain management; however, neither patient required use of narcotics during the course of medical care.^[30]^ Instead, over-the-counter ibuprofen (200–600 mg every 4–6 hours) sufficiently managed any injury-related discomfort. Additionally, there were no signs of treatment-related infection or adverse events documented with the use of placental allograft in either patient, which confirms literature demonstrating reduction in rates of infection with amnion application.^[27–29]^

The results presented in the aforementioned cases detailing the incorporation of HPMs in burn management may offer a new approach to outpatient burn management. The ease of application and associated clinical benefits of vCPM use may mitigate several of the complications seen post burn injury leading to favorable patient outcomes, even in the outpatient clinical setting.
